# Safety assessment of temozolomidee: real-world adverse event analysis from the FAERS database

**DOI:** 10.3389/fphar.2025.1578406

**Published:** 2025-08-06

**Authors:** Yu Liu, Lan Ma, Xiaojia Fu, Yi Zhang, Jinyu Zheng, Zhongjun Chen

**Affiliations:** ^1^ Department of Neurosurgery, Affiliated Huaian Hospital of Xuzhou Medical University, Huaian, China; ^2^ Department of Neurosurgery of Xuzhou Medical University, Xuzhou, China

**Keywords:** adverse event, FAERS database, glioma, chemotherapeutics, temozolomidee

## Abstract

**Background:**

Temozolomidee (TMZ) is an alkylating antitumor drug used in the treatment of glioblastoma and anaplastic astrocytoma. It is often combined with radiotherapy and has cytotoxic effects on tumor cells. Although temozolomidee has a certain efficacy in the treatment of brain malignancies, its numerous adverse effects (AEs) suggest that its safety needs to be thoroughly evaluated.

**Methods:**

Based on data from the FDA Adverse Event Reporting System (FAERS) database, a retrospective pharmacovigilance study was conducted to evaluate temozolomide-related adverse events. Methods for identifying temozolomide-related AEs signals include taking a case/non-case approach. Specific detection algorithms also include report Odds ratio (ROR), Proportional Report ratio (PRR), Bayesian confidence propagation neural network (BCPNN), and multi-item Gamma-Poisson constrictor (MGPS).

**Results:**

Among 48,766,547 FAERS reports, 13,608 TMZ-related AEs were identified. Males (53.66%) and patients aged ≥45 years predominated. The most frequent outcomes were hospitalization (35.76%), death (22.79%), and serious AEs (34.24%). Hematologic toxicities dominated, with “blood and lymphatic system disorders” showing the strongest signal (ROR 5.94, 95% CI: 5.73–6.15; PRR 5.48). Notable PTs included *petechiae* (ROR 9.87), *hemiparesis* (ROR 9.36), and *platelet count decreased* (ROR 8.61). Unexpected AEs, such as *pulmonary embolism* (ROR 4.96) and *Pneumocystis jirovecii pneumonia* (ROR 7.09), were identified. Renal/metabolic disorders (e.g., hypernatremia) and neurotoxic events (e.g., seizures, ROR 6.19) also demonstrated significant signals.

**Conclusion:**

This large-scale analysis highlights TMZ’s association with severe hematologic, thromboembolic, and opportunistic infection-related AEs in real-world settings. While expected toxicities (e.g., myelosuppression) were confirmed, novel signals like pulmonary embolism and neurotoxicity warrant further investigation. Clinicians should prioritize hematologic monitoring, thromboprophylaxis in high-risk patients, and *Pneumocystis* prophylaxis during corticosteroid co-administration. Future studies should validate these signals through prospective trials and mechanistic research to optimize TMZ’s risk-benefit profile in glioma therapy.

## 1 Introduction

TMZ is a first-choice alkylating agent inducted as a gold standard therapy for glioblastoma multiforme and astrocytoma. A majority of patients do not respond to TMZ during the course of their treatment. Asian populations are similar to those in Europe and the United States, but elderly patients (≥70 years old) may adjust the dose due to tolerance. The use of TMZ should be combined with age and pathological type (such as anaplastic astrocytoma) in the treatment of pediatric glioma (Horbinski et al., 2023). TMZ combined with radiotherapy can prolong the median survival time of patients with glioblastoma from 12.1 months to 14.6 months (5-year survival rate of about 10%), but it needs to be combined with the degree of surgical resection (e.g., total resection vs. partial resection) (Stupp et al., 2005). Available studies suggest that female patients may have better tolerance to TMZ (with a slightly lower incidence of hematologic toxicity), but there is no significant sex difference in efficacy (Zeiner et al., 2022). The drug has strong anti-tumor activity and can quickly cross the human blood-brain barrier after oral administration, thus playing a significant anti-tumor effect (Zou et al., 2022). TMZ is rapidly converted to the active product MTIC (3-methyl - (triazine-1-) imidazole-4-formamide) at systemic physiological pH (Petrenko et al., 2022). The cytotoxic effect of MTIC is mainly manifested by alkylation of guanine 6th oxygen atom and 7th nitrogen atom on DNA molecule. It plays cytotoxic role by mismatch repair of methylated adduct (Yang et al., 2019).

Despite the widely recognized efficacy of TMZ in the treatment of glioma, its association with a series of adverse events (AEs) requires us to evaluate its safety (Wei et al., 2024). The AEs of this drug include nausea, vomiting, bone marrow suppression and liver and kidney function impairment (Shiba et al., 2023). The incidence and severity of adverse effects in different patients are affected by the dose, the frequency of administration, and a number of other patient factors (Baro et al., 2022).

To clarify the risks associated with TMZ in clinical application, in-depth study and analysis of its pharmacovigilance are needed. The results of clinical trials and observational studies on historical data provide scientific evidence for the safety of TMZ (McBain et al., 2021). However, the sources of such evidence and the patient populations they represent may not fully match the breadth of AEs observed in real-world Settings (Tunthanathip and Sangkhathat, 2020). It is entirely possible that some AEs are underestimated or ignored in the daily clinical response process, which reminds us that we need to improve and perfect the pharmacovigilance system (Wang et al., 2024).

The United States Food and Drug Administration (FDA) Adverse Event Reporting System (FAERS) is an important resource for clinical drug safety assessment (Tian et al., 2022). This system collects AEs provided by medical practitioners, patients and pharmaceutical industry (Zhang et al., 2023). The safety of TMZ in clinical application and TMZ-related AEs were analyzed through this database, so as to provide reference for the safe use of TMZ in clinical practice.

The core objective of this study was to perform an exhaustive analysis of TMZ-related AEs in the FAERS database to assess adverse events associated with TMZ. By leveraging the FAERS data resources, we aim to identify previously unrecognized safety issues, assess the incidence and severity of reported adverse events, and contribute new research information on the safety profile of TMZ in clinical use.

## 2 Methods

### 2.1 Data sources

**FIGURE 1:**
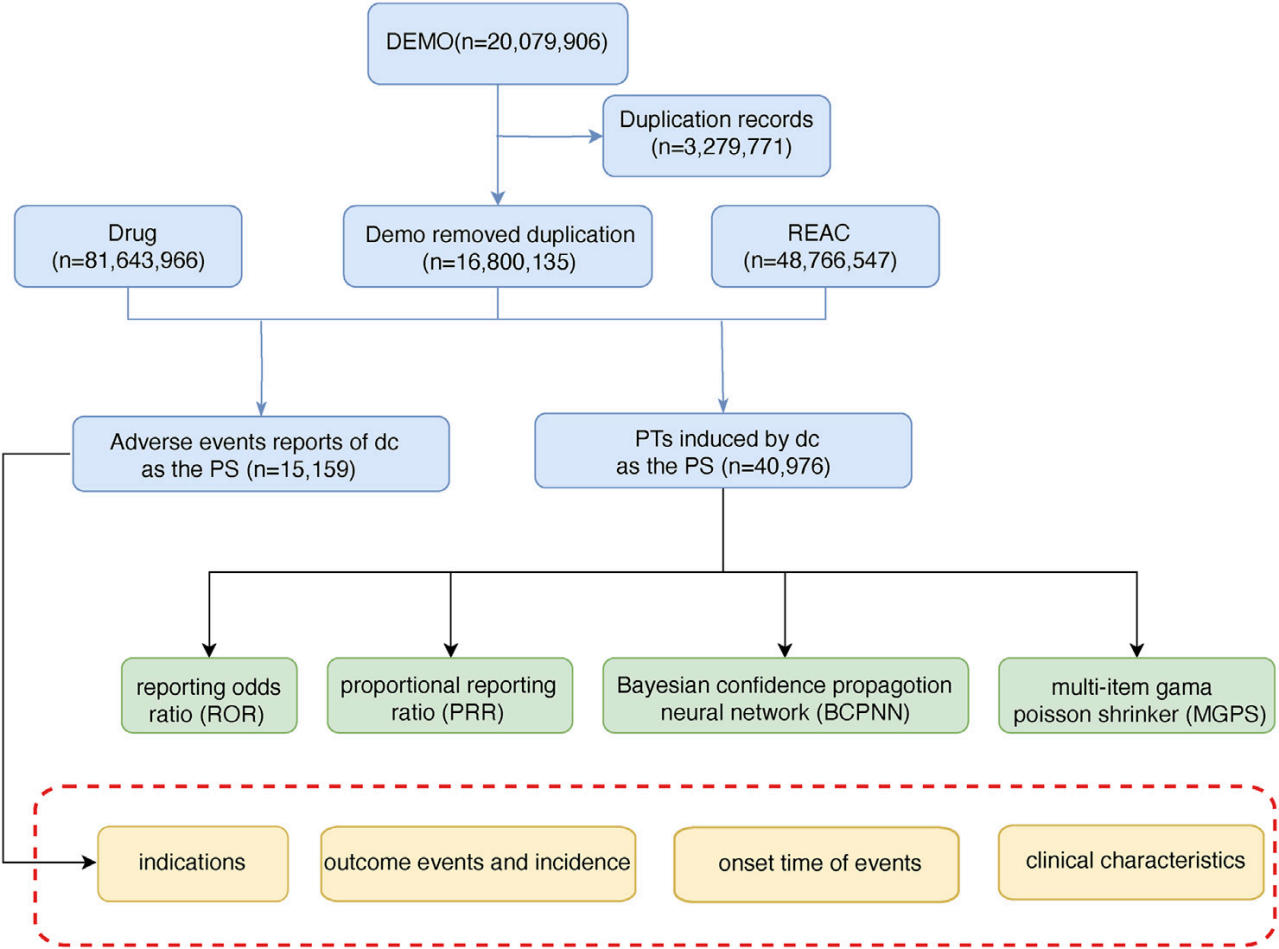
The flow chart of selecting TMZ-related AEs from FAERS database. (DEMO: demographic and administrative information; REAC: adverse drug reaction information; PS: primary suspected).

TMZ-related AEs from 2004 to 2023 were downloaded from the FAERS database (https://fis.fda.gov/extensions/FPD-QDE-FAERS/FPD-QDE-FAERS.html) in this study. According to ICH International Dictionary of Medical Terms, the PTS in AEs reports were systematically mapped and sinicized, and then mapped to the corresponding SOC to complete the harmonization of AEs international terminology. At the same time, doubtful and blank data, as well as ADE directly related to the indications, were deleted to form the original data of this study. The content contained basic information about the drug users, drug-related information, detailed descriptions of adverse effects, patient outcomes, sources of reports, duration of treatment, and reason for medication use or disease diagnosis. Strict duplicate removal procedures were followed according to FDA regulations to ensure the accuracy of the results and the credibility of the study conclusions. For this study, a “case” was defined as a cohort of patients who received TMZ and for whom TMZ-related AEs were documented. The combination of other drugs and AEs was defined as “non-case”. AEs not considered to be TMZ-related were also included in this study. The discovery of these AEs contributes to a comprehensive understanding of potentially significant safety issues with TMZ. Software and versions: The MedDRA term mapping tool (Version 24.1) was used for data standardization, R software (Version 4.2.3) was used for data cleaning and statistical analysis, and pharmaacoVigilance package was used for signal detection. The general flow chart of this study is as follows (Figure 1).

### 2.2 Data analysis methods

The reported inconsistency was analyzed based on traditional and Bayesian statistical methods, and the correlation between TMZ and AEs was obtained. In addition to reporting odds ratio (ROR), proportional reporting ratio (PRR), Bayesian confidence propagation neural network (BCPNN) and multi-item gamma Poisson constrictor (MGPS) were used. ROR and PRR were calculated using the following formula (a: the number of target AEs reports of the target drug; b: the number of other AEs reports of target drugs; c: the number of target AEs reports for other drugs; d: the number of other AEs reported for other drugs). n: total number (n = a + b + c + d) (Tables 1, 2). The generation of the AEs signal suggests a statistical association with the drug, with a higher value of the signal indicating a stronger association. Disproportionality analysis (such as ROR) is based on the overall report frequency of the database, aiming to screen potential signals, rather than causal inference. Subsequent multivariable analysis (such as logistic regression) can be used to control for confounding factors, but this study focused on signal detection and did not include control variables.

$$V\left(IC\right)=\frac{1}{{\left(\ln2\right)}^{2}}\{\left(\frac{C-C_{xy}+\gamma -\gamma_{11}}{\left(C_{xy}+\gamma_{11}\right)\left(1+C+\gamma \right)}\right)+\left(\frac{C-C_{x}+\alpha -\alpha_{1}}{\left(C_{x}+\alpha_{1}\right)\left(1+C+\alpha \right)}\right)+\left(\frac{C-C_{y}+\beta -\beta_{1}}{\left(C_{y}+\beta_{1}\right)\left(1+C+\beta \right)}\right)\}$$

$$\gamma =\gamma_{11}\frac{\left(C+\alpha \right)\left(C+\beta \right)}{\left(C_{x}+\alpha_{1}\right)\left(C_{y}+\beta_{1}\right)}$$

$$IC-2SD=E\left(IC\right)-2\sqrt{V\left(IC\right)}$$

$$\alpha_{1}=\beta_{1}=1;\alpha =\beta =2;\gamma_{11}=1;$$

$$C=a+b+c+d;C_{x}=a+b;C_{y}=a+c;C_{xy}=a$$

**TABLE 1:** Fourfold table of measures of disproportionality.

Item	Reports with the target AEs	All other AEs	Total
Reports with TMZ	a	b	a+b
All other drugs	c	d	c + d
Total	a+c	b + d	a+b + c + d

**TABLE 2:** Principle of dis-proportionality measure and standard of signal detection.

Algorithms	Calculation formula	Criteria
ROR	$ROR=\frac{a/c}{b/d}=\frac{ad}{bc}$ $95\%CI=e^{\ln\left(ROR\right)\pm 1.96\sqrt{\frac{1}{a}+\frac{1}{b}+\frac{1}{c}+\frac{1}{d}}}$	(1) a ≥3 (2) ROR ≥2 (3) 95%CI > 1
PRR	$PRR=\frac{a/\left(a+b\right)}{c/\left(c+d\right)}=\frac{a\left(c+d\right)}{c\left(a+b\right)}$ $\chi^{2}=\frac{{\left(\left|ad-bc\right|-\frac{n}{2}\right)}^{2}n}{\left(a+b\right)\left(a+c\right)\left(c+d\right)\left(b+d\right)}$	(1) a ≥3 (2) PRR ≥2 (3) χ^2^ ≥ 4 n = a + b + c + d
BCPNN	$E\left(IC\right)=\log_{2}\frac{\left(C_{xy}+\gamma_{11}\right)\left(C+\alpha \right)\left(C+\beta \right)}{\left(C+\gamma \right)\left(C_{x}+\alpha_{1}\right)\left(C_{y}+\beta_{1}\right)}$	(1) a ≥3 (2) IC-2SD > 0

## 3 Results

### 3.1 General characteristics

A total of 48,766,547 adverse drug reaction information (REAC) was retrieved from the FAERS database from 2004 through 2023 (Table 3). There were 13,608 reports of AEs associated with TMZ use. Among the reported cases, males accounted for 53.66% and females accounted for 46.34%. Most of them were middle-aged and elderly patients, and the proportion of patients aged 45 years and older was higher. The majority of reports were from the United States (50.44%), indicating high TMZ usage or robust reporting systems in this region. The outcome of relevant patients was mainly “hospitalization” (35.76%), and “other serious AEs” and “death” accounted for 34.24% and 22.79% of the outcomes, respectively. The vast majority of cases that reported detailed time to outcome (TTO) occurred within 2 months of TMZ use.

**TABLE 3:** Reporting characteristics of AEs signals for TMZ from Q1 2004 to Q4 2023.

Characteristics	Variable	Case number (pencentage)
sex	female	6306 (46.34)
	male	7302 (53.66)
age	<18	716 (5.26)
	18∼45	2058 (15.12)
	45∼65	4742 (34.85)
	65∼75	2327 (17.10)
	>=75	910 (6.69)
	unknown	2855 (20.98)
Reporter	Physician	4143 (30.45)
	Pharmacist	3759 (27.62)
	unknown	2098 (15.42)
	Other health-professional	1899 (13.96)
	Consumer	1693 (12.44)
	Registered Nurse	16 (0.12)
Reported countries	United States	6864 (50.44)
	other	4066 (29.88)
	Japan	514 (3.78)
	Canada	462 (3.40)
	France	423 (3.11)
	Germany	270 (1.98)
	United Kingdom	241 (1.77)
	Italy	212 (1.56)
	China	114 (0.84)
	Spain	110 (0.81)
	Australia	92 (0.68)
	Belgium	86 (0.63)
	Netherlands	82 (0.60)
	Brazil	72 (0.53)
Outcomes	hospitalization	4843 (35.76)
	other serious	4637 (34.24)
	death	3087 (22.79)
	life threatening	709 (5.23)
	disability	219 (1.62)
	required intervention to Prevent Permanent Impairment/Damage	37 (0.27)
	congenital anomaly	12 (0.09)
TTO(day) (Time to outcome)	<7	694 (7.76)
	7∼28	1204 (13.46)
	28∼60	1248 (13.96)
	>=60	1668 (18.65)
	unknown	4128 (46.16)

### 3.2 SOCs composition corresponding to TMZ-related AEs signal values

We identified SOCs with high reporting frequency by examining the pharmacovigilance of TMZ over the last 20 years (Table 4). The results showed that “blood and lymphatic system disorders” was the most prominent SOC, with 3481 reported cases and the strongest safety signal: the Relative Odds Ratio (ROR) was 5.94 (95% CI: 5.73–6.15) and the Proportional Reporting Ratio (PRR) was 5.48 (95% CI: 5.27–5.7), indicating a robust association with TMZ. This SOC also exhibited a markedly high Chi-square statistic (12905.25), an Information Component (IC) of 2.45 (95% CI: 2.4), and an Empirical Bayes Geometric Mean (EBGM) of 5.46 (95% CI: 5.3), further validating the strength of this signal. Other notable SOCs included “neoplasms benign, malignant and unspecified” (ROR 2.47, PRR 2.38) and “hepatobiliary disorders” (ROR 2.02, PRR 2.01), both of which demonstrated statistically significant associations with TMZ and warrant clinical attention. “Congenital, familial and genetic disorders” (ROR 1.77, PRR 1.76) and “investigations” (ROR 1.61, PRR 1.55) also showed positive signals, though with lower magnitude. In contrast, several SOCs such as “psychiatric disorders” (ROR 0.35, PRR 0.37), “eye disorders” (ROR 0.32, PRR 0.33), and “cardiac disorders” (ROR 0.41, PRR 0.42) exhibited ROR and PRR values < 1, indicating a weaker or absent association with TMZ.

**TABLE 4:** SOCs of TMZ-related AEs from FAES database.

SOCs	Case Reports	ROR (95% CI)	PRR (95% CI)	chisq	IC(IC025)	EBGM (EBGM05)
blood and lymphatic system disorders	3481	5.94 (5.73, 6.15)	5.48 (5.27, 5.7)	12905.25	2.45 (2.4)	5.46 (5.3)
neoplasms benign, malignant and unspecified	2422	2.47 (2.37, 2.58)	2.38 (2.29, 2.48)	1981.15	1.25 (1.19)	2.37 (2.29)
hepatobiliary disorders	711	2.02 (1.88, 2.18)	2.01 (1.86, 2.17)	361.12	1 (0.9)	2 (1.88)
congenital, familial and genetic disorders	182	1.77 (1.53, 2.04)	1.76 (1.53, 2.02)	60.1	0.82 (0.61)	1.76 (1.56)
investigations	3805	1.61 (1.56, 1.66)	1.55 (1.49, 1.61)	788.09	0.63 (0.58)	1.55 (1.5)
metabolism and nutrition disorders	1159	1.37 (1.29, 1.45)	1.36 (1.28, 1.44)	113.14	0.44 (0.36)	1.36 (1.3)
infections and infestations	2641	1.31 (1.26, 1.36)	1.29 (1.24, 1.34)	180.02	0.37 (0.31)	1.29 (1.25)
endocrine disorders	112	1.13 (0.94, 1.36)	1.13 (0.95, 1.35)	1.75	0.18 (-0.09)	1.13 (0.97)
nervous system disorders	3620	1.07 (1.03, 1.11)	1.06 (1.02, 1.1)	15.32	0.09 (0.04)	1.06 (1.03)
general disorders and administration site conditions	6754	1 (0.98, 1.03)	1 (0.98, 1.02)	0.12	0.01 (-0.03)	1 (0.98)
gastrointestinal disorders	3301	0.97 (0.94, 1.01)	0.98 (0.94, 1.02)	1.93	−0.03 (-0.08)	0.98 (0.95)
vascular disorders	757	0.88 (0.82, 0.95)	0.88 (0.81, 0.95)	12.01	−0.18 (-0.28)	0.88 (0.83)
respiratory, thoracic and mediastinal disorders	1629	0.85 (0.81, 0.89)	0.86 (0.83, 0.89)	41.07	−0.22 (-0.29)	0.86 (0.82)
injury, poisoning and procedural complications	2669	0.77 (0.74, 0.8)	0.79 (0.76, 0.82)	163.7	−0.34 (-0.4)	0.79 (0.76)
skin and subcutaneous tissue disorders	1270	0.59 (0.56, 0.62)	0.6 (0.57, 0.64)	355.53	−0.73 (-0.81)	0.6 (0.57)
renal and urinary disorders	384	0.54 (0.49, 0.6)	0.55 (0.5, 0.61)	146.52	−0.87 (-1.01)	0.55 (0.5)
immune system disorders	218	0.52 (0.45, 0.59)	0.52 (0.45, 0.6)	97.39	−0.94 (-1.13)	0.52 (0.47)
cardiac disorders	449	0.41 (0.38, 0.45)	0.42 (0.38, 0.46)	368.07	−1.25 (-1.38)	0.42 (0.39)
psychiatric disorders	847	0.35 (0.33, 0.38)	0.37 (0.35, 0.39)	973.13	−1.44 (-1.54)	0.37 (0.35)
eye disorders	256	0.32 (0.28, 0.36)	0.33 (0.29, 0.37)	364.08	−1.62 (-1.79)	0.33 (0.29)

### 3.3 Analysis of PTs for TMZ-Related AEs

**FIGURE 2:**
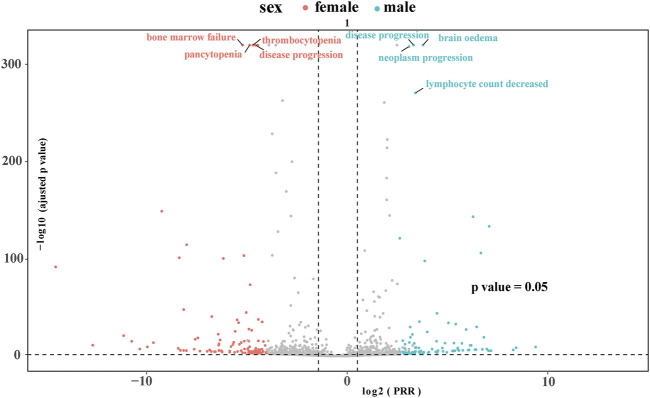
Volcano plot visualizing risk signals for TMZ-related adverse events. The X-axis represents log2-transformed proportional reporting ratios (log2PRR); values > 0 indicate a stronger association with TMZ. The Y-axis represents −log10-transformed adjusted p-values; higher values indicate greater statistical significance. The dashed horizontal line denotes the significance threshold (p = 0.05). AEs in the upper right quadrant (log2PRR >0 and −log10P-adjusted >1.3) are statistically significant signals.

At the PTs level, key TMZ-related AEs with significant signals included petechiae (ROR 9.87, 95% CI: 7.77–12.53), the strongest signal supported by high Chi-square (536.66) and IC (3.29); hemiparesis (ROR 9.36, 95% CI: 7.78–11.27) with robust associations (Chi-square 834.53, IC 3.21); and hematologic toxicities such as platelet count decreased (ROR 8.61) and febrile neutropenia (ROR 7.40). Unexpected AEs included Pneumocystis jirovecii pneumonia (ROR 7.09, 95% CI: 5.18–9.72, Chi-square 202.74) and pulmonary embolism (ROR 4.96, 95% CI: 4.44–5.53, Chi-square 1007.83), highlighting infectious and thromboembolic risks. Neurotoxic events like seizure (ROR 6.19) and aphasia (ROR 4.93), as well as hematologic AEs such as myelodysplastic syndrome (ROR 5.77) and agranulocytosis (ROR 5.16), also showed significant signals, collectively reflecting a spectrum of TMZ-related AEs including expected hematologic toxicities, unexpected complications, and neurotoxic manifestations. Figure 2 shows the volcano plot representing all the AEs associated with TMZ. AE signals in the plot’s right upper quadrant indicate a higher likelihood of occurrence during TMZ treatment. The 10 types of TMZ-related adverse events with the highest number of reports can be found in Supplementary Figure S1.

## 4 Discussion

The findings from this large-scale pharmacovigilance analysis of temozolomidee (TMZ)-related adverse events (AEs) provide critical insights into the drug’s safety profile in real-world clinical settings. By leveraging the FAERS database, this study identified both expected and unexpected AEs associated with TMZ, underscoring the importance of continuous monitoring and mechanistic exploration to optimize patient care.

The most prominent AEs observed in this study were hematologic toxicities, particularly thrombocytopenia, neutropenia, and febrile neutropenia, which align with TMZ’s known myelosuppressive effects. The high ROR and PRR values for these events (e.g., platelet count decreased: ROR 8.61, PRR 8.49) reflect TMZ’s alkylating mechanism, which disrupts DNA replication in rapidly dividing cells, including hematopoietic precursors. Hematologic adverse events (HAE) are common during glioblastoma (GBM) treatment and are usually associated with TMZ (Garcia et al., 2022). The identification of myelodysplastic syndrome (ROR 5.77) as a significant AE further raises concerns about long-term clonal hematopoiesis risks, a phenomenon increasingly recognized in alkylating agent therapy (Scaringi et al., 2013; Graubert, 2010). Women may have lower rates of hematologic toxicity (e.g., thrombocytopenia) due to the protective effect of estrogen on bone marrow hematopoiesis. In addition, sex-related differences in drug metabolism (such as CYP450 enzyme activity) may affect TMZ clearance, but more mechanisms are needed to support this (Scaringi et al., 2013).

Notably, the study uncovered unexpected AEs such as pulmonary embolism (ROR 4.96) and *Pneumocystis jirovecii* pneumonia (ROR 7.09). While TMZ is not classically associated with thromboembolic events, emerging evidence suggests that glioblastoma itself induces a hypercoagulable state due to tissue factor overexpression, and TMZ may exacerbate this risk by promoting endothelial dysfunction. For Pneumocystis jirovecii pneumonia (PjP) prophylaxis, the 2023 ESMO guideline recommends initiation when TMZ is combined with dexamethasone at a daily dose ≥4 mg for ≥2 weeks (due to increased risk of lymphopenia-induced immunosuppression). Our FAERS data show a strong signal for PjP in TMZ-treated patients (ROR 7.09, 95% CI: 5.18–9.72), with 39 reported cases—17 (43.6%) of which involved concurrent dexamethasone use (daily dose ≥2 mg, duration ≥1 week). This suggests that PjP risk may emerge even with lower corticosteroid doses or shorter durations than currently recommended, supporting consideration of lowering the threshold to dexamethasone ≥2 mg/day for ≥1 week in TMZ-treated patients.

For thromboprophylaxis, the NCCN Guidelines (Version 2.2024) recommend prophylactic anticoagulation in glioblastoma patients with a Khorana score ≥2 (risk factors including prechemotherapy platelet count >350 × 10^9^/L, hemoglobin <10 g/dL, or BMI ≥35 kg/m^2^) (Horbinski et al., 2023). Our FAERS data identified 323 pulmonary embolism cases (ROR 4.96, 95% CI: 4.44–5.53), with 102 (31.6%) occurring in patients with Khorana score 1 (single risk factor: e.g., hemoglobin 10–11 g/dL). This signal suggests that even patients with lower Khorana scores (1) may benefit from thromboprophylaxis during TMZ treatment, supporting potential adjustment of the threshold to Khorana score ≥1 in this population (Kapteijn et al., 2023).

The strong association between TMZ and neurotoxic AEs, such as hemiparesis (ROR 9.36) and seizures (ROR 6.19), warrants further investigation. While these events may reflect disease progression in glioma patients, TMZ’s potential to cross the blood-brain barrier and induce direct neuronal toxicity cannot be discounted. Preclinical studies have shown that TMZ metabolites, such as MTIC, may impair mitochondrial function in neurons, leading to oxidative stress and apoptosis. However, clinical data remain conflicting. No studies have found significant correlation between TMZ exposure and seizure frequency, suggesting that tumor-related factors (e.g., peritumoral edema) may dominate. This discrepancy underscores the need for biomarker-driven studies to distinguish drug-induced neurotoxicity from tumor-associated complications. TMZ has a short half-life (1.8 h) and requires daily administration, which may lead to persistent DNA alkylation and increase the cumulative risk of myelosuppression. A short half-life may also make drug concentrations highly volatile, inducing sudden toxicity (e.g., endothelial injury associated with pulmonary embolism) (Petrenko et al., 2022).

The renal and metabolic disorders identified in this study, including hypernatremia and hyperchloremia, may be linked to TMZ’s interaction with osmotic agents like mannitol, often co-administered to reduce cerebral edema in glioma patients. Mannitol-induced electrolyte imbalances are well-documented (Yuen et al., 2022; Mishra et al., 2023; Büyükkaragöz and Bakkaloğlu, 2023), but TMZ’s role in exacerbating these effects remains unclear. Studies have shown that TMZ may impair renal tubular function, reducing electrolyte excretion (Athavale et al., 2021). This hypothesis requires validation through dedicated renal safety trials, particularly in patients with pre-existing kidney disease.

While this study provides valuable real-world evidence, several limitations must be acknowledged. First, FAERS data are subject to underreporting and reporting bias, particularly for non-serious AEs. Second, the lack of granular clinical data (e.g., TMZ dosing schedules, concomitant medications) limits causal inference. For example, the high incidence of pulmonary embolism could be confounded by the widespread use of bevacizumab in glioma therapy, a known pro-thrombotic agent (Kanbayashi et al., 2022; Boileve et al., 2022). Future studies integrating FAERS with electronic health records (EHRs) or cancer registries could enhance signal validity.

Additionally, the study’s focus on disproportionality analysis does not establish causality. Prospective pharmacovigilance initiatives, such as the European Union’s PASS (Post-Authorization Safety Studies), are critical to confirm these signals. For instance, the signal for compartment syndrome (not listed in Table 5 but mentioned in the abstract) remains enigmatic and may represent a rare idiosyncratic reaction requiring case-level adjudication.

**TABLE 5:** Top 20 unexpected AEs of TMZ at PTs level.

PTs	Case reports	ROR (95% CI)	PRR (95% CI)	chisq	IC(IC025)	EBGM(EBGM05)
petechiae	68	9.87 (7.77, 12.53)	9.85 (7.79, 12.46)	536.66	3.29 (2.95)	9.78 (8.01)
hemiparesis	113	9.36 (7.78, 11.27)	9.33 (7.82, 11.13)	834.53	3.21 (2.95)	9.27 (7.94)
platelet count decreased	581	8.61 (7.93, 9.35)	8.49 (7.85, 9.18)	3820.52	3.08 (2.96)	8.44 (7.88)
hemiplegia	48	8.19 (6.17, 10.88)	8.18 (6.22, 10.76)	300.57	3.02 (2.62)	8.13 (6.41)
febrile neutropenia	280	7.4 (6.57, 8.32)	7.35 (6.53, 8.27)	1527.69	2.87 (2.7)	7.31 (6.62)
therapy partial responder	37	7.11 (5.15, 9.83)	7.1 (5.19, 9.72)	192.95	2.82 (2.36)	7.07 (5.39)
pneumocystis jirovecii pneumonia	39	7.09 (5.18, 9.72)	7.09 (5.18, 9.7)	202.74	2.82 (2.37)	7.05 (5.42)
neutrophil count decreased	161	6.38 (5.46, 7.45)	6.36 (5.44, 7.44)	723.5	2.66 (2.44)	6.33 (5.56)
seizure	399	6.19 (5.6, 6.83)	6.13 (5.56, 6.76)	1707.58	2.61 (2.47)	6.1 (5.62)
leukopenia	182	5.94 (5.13, 6.87)	5.92 (5.16, 6.79)	740.22	2.56 (2.35)	5.89 (5.21)
myelodysplastic syndrome	51	5.77 (4.38, 7.6)	5.76 (4.38, 7.58)	199.81	2.52 (2.13)	5.74 (4.56)
mental status changes	105	5.56 (4.59, 6.74)	5.55 (4.56, 6.75)	390.08	2.47 (2.19)	5.53 (4.71)
neutropenia	396	5.3 (4.8, 5.85)	5.25 (4.76, 5.79)	1359.08	2.39 (2.24)	5.23 (4.81)
agranulocytosis	59	5.16 (4, 6.67)	5.16 (4, 6.66)	196.88	2.36 (2)	5.14 (4.15)
pulmonary embolism	323	4.96 (4.44, 5.53)	4.92 (4.37, 5.53)	1007.83	2.3 (2.14)	4.91 (4.48)
aphasia	103	4.93 (4.06, 5.98)	4.92 (4.04, 5.99)	320.41	2.29 (2.02)	4.9 (4.17)
product dispensing error	48	4.9 (3.69, 6.5)	4.89 (3.72, 6.43)	148.05	2.29 (1.88)	4.88 (3.85)
disseminated intravascular coagulation	45	4.6 (3.44, 6.17)	4.6 (3.43, 6.17)	126.34	2.2 (1.78)	4.59 (3.59)
hepatic function abnormal	104	4.52 (3.72, 5.48)	4.51 (3.71, 5.49)	282.88	2.17 (1.89)	4.49 (3.82)
white blood cell count decreased	325	4.47 (4.01, 4.99)	4.44 (3.95, 4.99)	865.68	2.15 (1.99)	4.43 (4.04)

## 5 Conclusion

This analysis reinforces the need for vigilant monitoring of TMZ-treated patients, particularly for hematologic, thromboembolic, and opportunistic infections. Clinicians should consider: (1) routine blood counts to monitor hematologic toxicity; (2) thromboprophylaxis in patients with a Khorana score ≥1 (aligning with our FAERS signal of pulmonary embolism in lower-risk groups, beyond the current NCCN threshold of ≥2 (Horbinski et al., 2023)); and (3) Pneumocystisprophylaxis when TMZ is combined with dexamethasone ≥2 mg/day for ≥1 week (supporting a lower threshold than the ESMO-recommended ≥4 mg/day for ≥2 weeks, based on our PjP signal). For unexpected AEs like compartment syndrome, heightened clinical suspicion and reporting are essential to clarify their association with TMZ. Future research should prioritize mechanistic studies to elucidate TMZ’s role in neurotoxicity and renal dysfunction, as well as randomized trials evaluating risk mitigation strategies (e.g., dose optimization, supportive therapies). By integrating real-world data with preclinical models, the oncology community can refine TMZ’s safety profile and improve outcomes for glioma patients.

## Data Availability

The original contributions presented in the study are included in the article/Supplementary Material, further inquiries can be directed to the corresponding authors.
