# Supplementary figures and images for: Safety assessment of temozolomidee: real-world adverse event analysis from the FAERS database
Source: Front Pharmacol. 2025 Aug 6;16:1578406. doi: 10.3389/fphar.2025.1578406 (PMC12365615; doi:10.3389/fphar.2025.1578406)

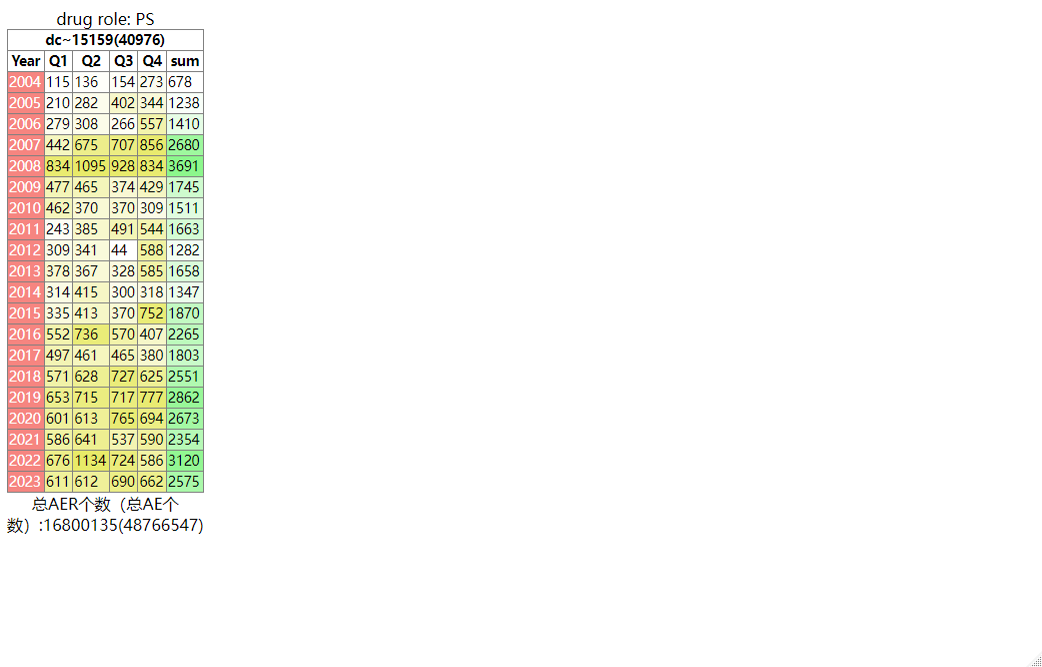

Supplement: Supplementary file 1 [file Image1.png]
